# Environmental Regulations and Corporate Green Innovation in China: The Role of City Leaders’ Promotion Pressure

**DOI:** 10.3390/ijerph18157774

**Published:** 2021-07-22

**Authors:** Le Yang, Jiahao Zhang, Yufeng Zhang

**Affiliations:** 1 Department of Public Finance, School of Public Finance and Taxation, Central University of Finance and Economics, Beijing 100081, China; 2019212070@email.cufe.edu.cn; 2 Department of Administration, College of Philosophy, Law& Political Science, Shanghai Normal University, Shanghai 201418, China; 1000479249@smail.shnu.edu.cn; 3 Department of Finance, Finance and Economics School, Jimei University, Xiamen 361031, China

**Keywords:** environmental regulations, green innovation, city leaders’ promotion pressure, corporate innovation

## Abstract

China and other emerging market countries have suffered from the problem of environmental pollution while developing rapidly in the past few decades. In recent years, many countries have introduced strict environmental regulations in order to achieve sustainable development. This paper discusses the relationship between environmental regulations and corporate green innovation from the perspective of regional leaders’ promotion pressure. The empirical results show that direct policy regulation within the region stimulates green innovation on the part of enterprises, and the promotion pressure of city leaders has a further positive moderating effect on the positive correlation between environmental regulations and enterprises’ green innovation. The conclusion of the study proves that a strict environmental policy can promote the effectiveness of an environmental performance appraisal system in the sustainable development plans of cities and enterprises. This paper not only reveals the influence path of official promotion pressure on the sustainable development of enterprises in the administrative area from the micro perspective but also sheds some light that may improve government governance and promote the transformation of enterprises.

## 1. Introduction

China, as an emerging market country, needs to quickly address its backward economic situation. China chose a rough and unsustainable route of development in the early stage of its economic development [1]. In the early stage of the reform and opening-up that began in the 1970s, various regions of China blindly pursued economic benefits, taking GDP as the most important factor for assessing the success of a government administration. Such unsustainable development (blindly pursuing economic benefits) has had an irreversible impact on the environment [2]. With the continuous growth of China’s economy, this unsustainable growth mode of sacrificing the environment for the economy is no longer sustainable [3,4]. Especially in the past 10 years, the environmental pollution problem represented by PM2.5 pollution has been unsolvable [5], which has brought about dissatisfaction for Chinese residents. Therefore, the Chinese central government is paying more and more attention to sustainable development that balances the environment and the economy [6].

The 18th National Congress of the Communist Party of China, held in Beijing in November 2012, set the guiding ideology for China’s future economic and social development. At this meeting, eight important programs were formed, which will serve to guide strategic thinking about China’s future development. Two of them are: “vigorously promote the construction of an ecological civilization and reverse the trend of deterioration of the environment” and “implement the strategy of innovation-driven development”. Since then, the concept of sustainable development, such as environmental protection and enterprise innovation, has become the guiding policy of governments at all levels in China.

In March 2006, *The Outline of the 11th Five-Year Plan for National Economic* and *Social Development of the People’s Republic of China* for the first time set environmental standards, such as total emissions of chemical oxygen demand and sulfur dioxide, two major pollutants, and limited the energy consumption per unit of GDP, making these binding indicators for the performance appraisal of local officials. Since then, environmental assessment has been formally incorporated into the political performance assessment system for local leaders. In recent years, environmental governance achievements and targeted poverty alleviation achievements have become the two most important indicators for the promotion and assessment of Chinese officials [7,8]. The environmental performance, especially the green innovation performance of enterprises in the area where the officials are located, is now a top priority in the assessment of officials. Green innovation refers to innovation activities related to technology or products in the fields of energy conservation, environmental protection, and resource recycling, which is one of the most important means for enterprises to achieve sustainable development [9]. It reflects the strategic needs of enterprises in various aspects, such as the pursuit of technological innovation [10], the fulfillment of corporate social responsibility [11], transformation, and upgrading [12]. China’s central and local governments are trying to encourage companies to invest more in environmental protection and increase green innovation. On the one hand, they provide a lot of policy support in terms of taxation, government financial subsidies, managers’ evaluation of excellence, the promotion of leaders of state-owned enterprises, etc. On the other hand, penalties will be levied against enterprises that are not working towards environmental protection and do not invest actively in green innovation. Every winter, a large number of enterprises are ordered to stop production because the environmental protection technology transformation is not up to standard. Especially in smoggy areas, local officials are often criticized by Communist Party committees and governments at higher levels for failing to implement environmental protection efforts and for the slow pace of enterprises in their areas in phasing out outdated production capacity (high-pollution production methods), undermining the political prospects of city leaders.

The government’s environmental regulation policies can significantly affect the governance decisions of enterprises [13,14]. However, the choice of strategy can significantly affect the environmental regulations [15]. China’s government governance is a typical “M-shaped” governance structure [16]—that is, each region adopts a “block economy” with a similar structure and function. The higher-level government is highly centralized, while the lower-level government retains a large degree of local decentralization. Specifically, China’s environmental regulation policies are formulated by the central government and then implemented by local governments. In the process of implementation, local governments formulate regulations according to the specific conditions of their respective regions. However, local officials can also choose a looser or stricter implementation of environmental regulation policies based on various factors [3]. Based on the above analysis, this paper draws the following basic logic chain: first, strict local government environmental governance policies increase the compliance cost of enterprises, force enterprises to innovate technology, eliminate backward production modes, and thus promote green innovation of enterprises. Secondly, as the assessment of regional leaders by superior leaders focuses most on environmental governance and economic construction, the promotion pressure on urban leaders will intensify the stimulating effect of environmental regulations on the green innovation of enterprises.

The unique promotion and assessment rules for Chinese officials provide an interesting and meaningful research angle for the study of external governance issues of corporate governance. Therefore, from the perspective of promotion pressure of city leaders, this paper discusses the relationship between environmental regulations and the green innovation decisions of enterprises, as well as external factors. After theoretical analysis and discussion, this paper proposes two research hypotheses: “environmental regulation promotes the green innovation of enterprises” and “the pressure on city leaders increases their promotion of environmental regulations and green innovation for enterprises”.

To verify the basic assumptions of this paper, this paper takes China’s A-share listed companies from 2010 to 2019 as samples and makes an empirical study using relevant data. The baseline regression results of this paper show that environmental regulations do promote the green innovation of enterprises. To enhance the robustness of the empirical results, three variables were used to conduct robustness tests: environmental regulation (ERS) was delayed by one period; we changed the measurement method for green innovation; and we changed the measurement method for environmental regulations. The robustness test results were consistent with the baseline regression results. Considering the potential problem of endogeneity, this paper further controls the intersection term between industry and year to alleviate the possible problem of endogeneity in a time series. The annual precipitation at the enterprise’s location is used as a tool variable to carry out a Two Stage Least Square (2SLS) regression. After controlling for the problem of endogeneity, the results of the baseline regression in this paper are still robust.

This paper further discusses the moderating effect of urban leaders’ promotion pressure on environmental regulations and green corporate innovation. The empirical results show that the promotion pressure of city leaders has a further positive moderating effect on the direct positive correlation between environmental regulations and the green innovation of enterprises. To control the problems of endogeneity that may be caused by the regulation effect, the Instrumental Variables-Two Stage Least Square (IV-2SLS) method with interaction terms was used in this paper [17] to control problems of endogeneity. The average annual precipitation in the place where the enterprise is located and the duration of the previous mayor (tenure) were used as instrumental variables of environmental regulations and the promotion pressure of city leaders, respectively. After controlling for the problem of endogeneity, the positive moderating effect was still maintained.

The possible academic contributions of this paper are as follows: (1) Based on the government system of countries in transition, it deepens the research on enterprise innovation. The vast majority of enterprises’ (green) innovation is based on the microscopic perspective of enterprises [18,19,20,21,22]. In this paper, the impact of the external environment on enterprise innovation is examined from the level of local officials’ demands for the political performance of a more in-depth investigation. It is helpful to understand this is a key to economic growth and optimization. (2) The theory of political competition is enriched from the perspective of the sustainable development of enterprises. Although some studies have discussed this issue from the perspective of official tenure [23], aside from the general defects of empirical studies, the practice of simply using the length of tenure without considering economic factors may be slightly superficial. Based on the promotion pressure of local leaders, this paper carefully discusses the cause-and-effect identification problem and then makes a more scientific discussion of this problem. (3) It proves the effectiveness of the environmental performance appraisal system in environmental governance from the micro-perspective of enterprises. The micro-sustainable governance of enterprises and the regional sustainable governance are mutually dependent, and they can avoid conflicts and develop together.

The rest of this paper is arranged as follows: Section 2 gives the background, theory, and hypothesis; Section 3 gives the methods; Section 4 gives the empirical results; Section 5 addresses further research topics, and Section 6 is the discussion and conclusions.

## 2. Background, Theory, and Hypothesis

### 2.1. Environmental Regulations and Green Innovation

For a decade or more, haze has been the most serious environmental problem in China, and it remains a social issue that Chinese people are concerned about. The problem of haze has greatly affected the daily production of Chinese society, public health [17,18], and even people’s trust in government officials. In 2005, the State Council of China issued the “Decision on Implementing the Scientific Outlook on Development and Strengthening Environmental Protection”, which first put forward the concept of “environmental performance assessment”. Since then, environmental regulation has become an important issue for local officials. Since Chinese President Xi Jinping put forward the concept of sustainable development (“clear waters and lush mountains are invaluable assets”), every aspect of China’s production and life has been conscientiously implementing this instruction. China has formulated and revised a large number of laws and regulations related to environmental protection, has seriously investigated and punished a series of illegal behaviors of enterprises [20], and has encouraged enterprises’ green innovation behavior [21].

The environmental regulation system of Chinese governments at all levels has become more and more strict. The cost of breaking the rules is getting higher and higher. Enterprises are forced to carry out environmental protection activities and green innovation. On the other hand, the Chinese government has created a series of preferential policies, such as preferential government procurement, tax exemption, and an interest discount on loans, to encourage enterprises’ green innovation, and has vigorously cultivated citizens’ knowledge of green concepts to promote green consumption. All these factors have promoted green innovation by enterprises. Especially in recent years, many enterprises have adopted green innovative business models or have applied for green patents. Among them, Alibaba Group’s “Ant Forest Plan” is a well-known one that has accumulated a large amount of “carbon tax” income for the group, which has improved user stickiness and fulfilled the social responsibility of the enterprise. As another example, the Gree Group has improved air conditioning compressor technology, which not only cuts down environmental regulation costs but also protects the atmosphere. In short, China’s existing policies provide a better political and economic environment for the green innovation activities of enterprises.

Most theoretical studies on environmental regulations and the green innovation of enterprises are based on Porter’s Theory [22]. According to Porter’s Theory, reasonable environmental regulation policies will not impose unnecessary costs on enterprises but will promote enterprise innovation, improve production efficiency, and enhance the international competitiveness of enterprises [23]. Based on this view, it is essentially a mechanism of “innovation compensation”, which reflects the concept of the survival of the fittest [24]. The government’s strict environmental regulations are unavoidable. Enterprises will face severe administrative penalties if they fail to meet the government’s requirements on pollution and energy consumption. However, under China’s unique governance mode, combined with the party–government framework, enterprises can also receive compensation for green innovation behaviors. Enterprises can reap economic benefits, and managers may also obtain political capital and social honor. China’s mainstream media are all managed by the propaganda department of the communist party of China, which has rich experience in ideological and political education and propaganda. With the improvement of the government’s requirements on environmental protection [25], the mainstream media have gradually increased their publicity of environmental protection concepts, which has awakened consumers’ environmental awareness [26,27] and caused them to have higher requirements for enterprises’ green production. Enterprises may struggle to meet the environmental regulations and consumer requirements for their original product line. This element of consumer choice will be further intensified by the mainstream media. High-pollution and high-energy-consuming products or consumer goods are being gradually phased out in response to consumer demand and the loss of the market. This also forces enterprises to launch “innovation compensation mechanisms” [28,29] and carry out innovation activities. At the same time, green innovation can lead to more policy compensation, more tax relief, and fiscal payment transfer. In terms of government bidding and procurement, priorities have to be set. This can satisfy consumers’ preferences, better fulfill corporate social responsibility, and obtain publicity benefits and social recognition. Therefore, the impetus for green innovation of enterprises is greater than that for other innovation activities [30] The enhancement of environmental regulation intensity will increase the external incentives for the green innovation of enterprises. Mandatory emission standards to reduce the short-term impact will gradually become the norm for enterprises. For both new and existing enterprises, it is normal to allocate resources to carry out green innovation and then gradually complete the green transformation required by the government [3]. In addition, direct policy regulation will also attract the attention of the media [31], which will further guide the innovation resources of universities and research institutes into more environmentally friendly innovation activities. Therefore, Hypothesis 1 is proposed:

**Hypothesis** **1** **(H1).** *Environmental regulation promotes the green innovation of enterprises.*

### 2.2. Promotion Pressure and Environmental Regulation of Local Leaders

Under China’s current political governance system, there are four tiers of cities: provincial (municipality), subprovincial (there are 15 subprovincial cities in the People’s Republic of China, including Shenzhen, Xiamen, Ningbo, Qingdao, and Dalian, which are separately listed in the national plan, as well as 10 provincial capitals, in which the administrative rank of Party Secretary of the 15 subprovincial cities is subprovincial), prefecture-level cities, and county-level cities. The establishment of county-level cities is mostly outside of the consideration of economic development, as they are basically managed by prefecture-level cities, which are essentially county-level administrative units. Therefore, this study does not discuss the county level. In most cases, the rank of a city determines the position of local leaders in the region [32]. The assessment and selection of local officials are mainly carried out by higher organizations under the leadership of the Communist Party of China. The selection and assessment of local officials are influenced by many factors, such as the reputation of the government, economic growth, Party achievements, the completion of special tasks (such as targeted poverty alleviation and disaster relief), age, educational background, and so on. Officials in China serve five-year terms, with a maximum of 10 years in the same position. However, most city leaders do not last more than 10 years in office. From 2000 to 2011, the average tenure of urban leaders in China was only 3.7 years [33]. From 2012 to 2019, Chinese city leaders averaged about 5.2 years in office, slightly longer than the five-year term, according to the preliminary statistics gathered for this paper. According to Chinese political custom, when city leaders reach retirement age, they are generally promoted to deputy leader of a higher-level body such as the People’s Congress or the Political Consultative Conference. Presumably, a city leader with promotion aspirations would not be content to serve two full terms but would try to accrue political capital while in office. However, the pressure to move up decreases with age.

As mentioned above, since 2006, the law-making bodies of the People’s Republic of China—the 11th five-year plan, the state environmental protection administration of China, the National Development and Reform Commission, the National Bureau of Statistics, the National Energy Bureau, and other departments—have promulgated a series of specific regulations that form a relatively complete system of environmental protection and the assessment of local officials. The main feature of the policy system is layers of decomposition and assessment of the environmental constraint index—namely, the central government sets overall control targets for total emissions of major pollutants and energy consumption per unit of GDP. The central government and provincial leaders sign a responsibility document for environmental protection targets, and the national targets are decomposed and implemented to the provinces, which then decomposes and implements the provincial targets to the prefecture-level cities. Cities will further allocate quotas to key polluters in districts, counties, and areas under their jurisdiction. Local officials at all levels are responsible for the energy conservation and emission reduction targets under their jurisdiction, and higher authorities assess the achievement of targets. The specific evaluation method for the central, provincial, and municipal levels is to send an inspection team regularly to the provinces, municipalities, counties (area), and key enterprises to assess environmental indicators, make a comprehensive assessment report, and announce it publicly. The results of the appraisal are taken to the government of the next level up to decide on the appointment or dismissal of cadres at all levels [34].

### 2.3. Moderation Effect of Official Promotion Pressure

The earliest theory related to officials and the local economy in China is “federalism with Chinese characteristics” [35], which believes that the institutional and economic basis of China’s economic growth is fiscal decentralization and administrative decentralization from the central government to local governments. Under this system, local government leaders have not only financial incentives but also other implicit incentives [36]. This system allows for implicit comparisons between the leaders of various cities, causing more “peer pressure”. In China, local leaders are not directly elected by citizens but appointed by the local Communist Party or people’s congresses after recommendation and vetting by higher-level organizations. In this process, the role of superior leaders is pivotal. In China, the local leaders in the administrative pyramid, besides paying attention to the local economic development, are naturally concerned about the opportunity of promotion, and this incentive may be a more important motive in reality. Such “promotion competition” will make local officials concerned about their careers and can cause them to pay more attention to the assessment of leaders [29,37,38]. The policy preferences of the central government greatly influence the governing choices of local leaders. The literature verifies that, during the period of rough economic development in China, due to the central government only assessing economic benefits and not paying attention to the environment, there is an obvious positive correlation between the promotion probability of local leaders and the GDP growth rate [39]. At the same time, the pollution problem is getting worse, and large areas of forests and lakes have been destroyed [40]. Some studies have verified that the promotion probability of local leaders is positively correlated with the GDP growth rate of the area [41], which provides empirical evidence for the existence of officials’ promotion incentives. In a previous paper, we mentioned that the Chinese government’s assessment of local leaders is no longer simply based on GDP, and that environmental protection is now almost as important as economic development. The hidden income brought about by environmental protection to local officials will encourage them to pay more attention to economic performance [42]. The basic theoretical logic chain of this relationship is as follows: in the context of “federalism with Chinese characteristics,” the promotion pressure brought about by horizontal comparisons reveals more implicit incentives, which are reflected in the promotion inspection principles of superiors. As a result, city leaders pay more attention to the preferences of the central government. Therefore, the influence of the ruling preference and policy orientation of the superior government on local leaders will be further amplified through promotion pressure. Based on the research objectives of this paper, the promotion pressure of city leaders will amplify the effect of environmental regulation policies on the green innovation of enterprises. Therefore, Hypothesis 2 is proposed:

**Hypothesis** **2** **(H2).** *The promotion pressure of city leaders enhances the effect of environmental regulation on the green innovation of enterprises*.

## 3. Methods

### 3.1. Data and Sample Selection

This paper took Chinese A-share listed companies from 2010 to 2019 as the sample. The following samples were removed: (1) ST, ST*, and enterprises with substantial changes in their main business. This was mainly based on the sustainability and continuity of the business. (2) Financial enterprises. This was because the accounting standards of financial enterprises differed greatly from those of other enterprises. Under China’s current accounting standards for listed enterprises, financial statements of financial enterprises were not directly comparable to those of other enterprises. (3) Enterprises that had not disclosed green innovation data or had disclosed green innovation data for less than 2 years. (4) Enterprises that were missing important data, including relevant regional statistical data on the location of the enterprise and on the enterprise itself. In this paper, statistics on 6813 individuals–years were obtained.

Unless otherwise stated, all regional statistical data in this paper were obtained from the Chinese Research Data Services Database (CNRDS) and the China Stock Market and Accounting Research Database (CSMAR). All the financial data of the companies were from the Wind financial terminal. To exclude the influence of outliers, we Winsorized all continuous variables at the 1% level in this paper.

#### 3.1.1. Explained Variable: Enterprise Green Innovation (Green_in)

In this paper, the number of enterprises authorized for green innovation was used as the proxy variable of enterprises’ green innovation. The specific screening steps were as follows:

(1) The patent data of all the listed companies in the China Research Data Service Platform (CNRDS) were taken as the baseline (due to the fine-tuning of the statistical caliber of green patents, data from 2017 to 2019 were adjusted by referring to the “green innovation” sub-database of the CSMAR database) and matched with a China Intellectual Property Classification Number (IPC). (2) We matched the obtained IPC number with the IPC number in the green patent classification database published by the International Intellectual Property Organization (WIPO) and eliminated nongreen patents; (3) we calculated the number of green patents issued to each company and took the logarithm after adding 1 (see Table 1).

#### 3.1.2. Explanatory Variable: Environmental Regulatory Strength (ERS)

The strength of environmental regulations reflects the strictness of the formulation and the implementation of environmental regulatory policies in a region [43,44], with obvious regional characteristics [45]. The measurement methods of environmental regulation were very diverse, but the comprehensive index method based on pollutant emission was most commonly used [46]. In this method, industrial wastewater, SO_2_, and smoke and dust in the whole country were used to calculate the environmental regulation strength in the region [47] (see Table 1).

#### 3.1.3. Moderating Variable: Official Promotion Pressure (off_pre)

In terms of official promotion pressure, competitive pressure was ultimately an indicator of economic performance. In this paper, comprehensive indicators, composed of the local government real GDP growth rate, the average wage growth rate of employees, and fiscal surplus, were adopted [48] (see Table 1).

#### 3.1.4. Control Variables

In this paper, control variables were selected from 3 perspectives: the basic characteristics of the city, the level of infrastructure construction and the individual enterprise, and the year and industry of the enterprise (controlled at the same time).

The urban basic characteristics, real GDP per capita (*PGDP*), degree of industrialization (*lnindout*), the proportion of tertiary industry *(thrind*), and fixed asset investment (*lninvest*) were selected.

Per capita real GDP represents the wealth of residents in a country or region [6], which is an important basis for enterprise innovation. The higher the per capita real GDP, the better the enterprise’s (green) innovation [49]. The degree of industrialization reflects the level of industrialization in a region and the environment in which enterprises produce their products. A higher level of industrialization will bring about an agglomeration effect, which will encourage enterprises to carry out green innovation activities to a certain extent [50]. The higher the proportion of the tertiary industry, the richer the industrial structure, and the higher the technological content [51,52], the better the innovation environment [53]. Fixed-asset investment activities of construction and the purchase of fixed assets reflect the relationships between the size, speed, and scale of investment in fixed assets. If we consider the direction of the comprehensive index, it largely reflects the region’s economic dynamism [54]; fixed-asset investment growth will, to a certain extent, promote enterprise innovation [5].

The level of infrastructure construction: in China, education, medical care, and communication were the 3 indicators used to measure the level of infrastructure construction in a region. Therefore, we selected the number of local basic education schools (*lnschool*) [54], the number of mobile phone users per 10,000 people at the end of the year (*lnmobile*) [3], and the number of III-A General hospitals in this region (the highest level in the hospital evaluation system of the Ministry of Health of China) (*lnhospital*) [55] as measures of the 3 indexes.

In research measuring education level, some scholars measured the average education level of residents [3], but this method reflects the level of local human resources more than the education level of a region. For example, Henan Province in China has a high level of education due to the high pressure of entering school. However, due to its backward economy, it is difficult for children to enter a more exclusive school, which leads to a lack of attraction of talent and a brain drain. The number of schools at or below high school in an area is a better indicator of the level of education because most of China’s regional brain drain occurs after university. For assessing the level of communication facilities, the length of regional long-distance optical cable lines is the most common factor. However, data were only available at the provincial level in China, not the city level. The logic behind this was that various communication tools are playing an increasingly important role in regional communication, and communication requires the region to provide long-distance optical cable facilities [3]. Mobile phones are the most widely used means of communication in China. Therefore, we believe that the number of mobile phone users per 10,000 people at the end of the year was also a reasonable way to measure the level of communication infrastructure in a region. In terms of measuring medical and healthcare, we chose to consider the number of III-A general hospitals in the region. Because of the extreme imbalance in China’s medical development, Henan, China’s most populous province, has fewer third-class hospitals than a single city in Sichuan province, Chengdu. For example, Xinyang City in Henan Province has a permanent population of 6.5 million, but there was only 1 III-A general hospital, and it had a serious shortage of hospital beds. Residents often must cross cities and provinces to have access to better medical resources. In Chengdu, Sichuan province, there were 42 III-A general hospitals, leaving a large number of high-quality medical resources idle. Therefore, the number of III-A general hospitals in a region can better reflect the medical level of the region.

The level of individual enterprise after surveying review articles on corporate governance and enterprise environmental governance [10,56,57,58], and other relevant studies, we selected the time of establishment (*establish*) [57], shareholding ratio of management *(share*) [56], shareholding ratio of the top shareholder (*top*) [58,59], and management scale (*drcnum*)[60], total assets (*lnassets*) [61], return on assets (*roa*) [62], an asset–liability ratio (*lev*) were also considered [63]. These 6 indicators were the control variables at the enterprise level, which can affect the innovation decisions of the enterprise. The establishment time of an enterprise reflects the reputation of the enterprise in its field [64], as well as the life cycle of the enterprise, to a certain extent [65]. Management shareholding ratio and management scale, to a large extent, reflect the concentration degree of corporate equity [66] and the transaction cost of corporate governance and significantly affect the innovation decisions of enterprises [67]. The total assets of an enterprise reflect the operating volume and economic strength of the company. Companies with a large economy have more capital to invest in innovation [59]. Return on assets reflects the operating performance of an enterprise [68]. A high return on assets indicates that an enterprise has achieved good results in terms of increasing income and saving funds [60]. Companies with better business performance tend to have higher levels of innovation [59]. The asset–liability ratio reflects the financial operating leverage and operating risk of an enterprise [64], and the financial choices of an enterprise will also affect the innovation of an enterprise.

See Table 3 for the specific calculation method of control variables.

### 3.2. The Empirical Model

To verify that environmental regulations can affect enterprises’ green innovation, referring to relevant studies [29,68], we established an empirical model as shown in Equation (1):

(1) $$\mathrm{green}\_{\mathrm{in}}_{i,t}=\beta_{0}+\beta_{1}{\mathrm{ERS}}_{i,t}+\beta_{2}{\mathrm{controls}}_{i,t-1}+\overset{n}{\underset{i=1}{\sum }}\mathrm{industry}+\overset{n}{\underset{i=1}{\sum }}\mathrm{year}+\varepsilon ,$$

where is the green innovation of the enterprise, ${\mathrm{ERS}}_{i,t}$ is the environmental regulation level of the city where the enterprise is registered, ${\mathrm{controls}}_{i,t-1}$ is the lag period of all the control variables, and $\sum_{i=1}^{n}\mathrm{industry}$ is a dummy variable. $\sum_{i=1}^{n}\mathrm{year}$ is the annual dummy variable and ε is the stochastic disturbance. It needs to be noted that, considering the actual situation of the enterprise’s operations, and in accordance with customary practice, all the control variables in this paper were delayed by a 1-year period. In order to reduce the influence of heteroscedasticity illumination, all regressions in this paper adopted clustered robust standard error.

To verify that promotion pressure of regional officials can change the impact of environmental regulations on the green innovation of enterprises, we established an empirical model as shown in Equation (2):

(2) $$\mathrm{green}\_{\mathrm{in}}_{i,t}=\beta_{0}+\beta_{1}{\mathrm{ERS}}_{i,t}+\beta_{2}{\mathrm{ERS}}_{i,t}{*\mathrm{off}}_{\mathrm{pre}}{}_{i,t}+\beta_{3}{\mathrm{controls}}_{i,t-1}+\overset{n}{\underset{i=1}{\sum }}\mathrm{industry}+\overset{n}{\underset{i=1}{\sum }}\mathrm{year}+\varepsilon ,$$

where $\mathrm{off}\_{\mathrm{pre}}_{i,t}$ is the promotion pressure of officials in the city where the enterprise is registered, and other symbols are as listed for Equation (1).

### 3.3. Methods of Endogeneity Test and Robustness Test

#### 3.3.1. Endogeneity Test Method

To control possible endogeneity problems, in this paper, we used 2 methods to discuss the endogeneity: controlling the change of industry over time and the instrumental variable method. The processes are as follows:

Controlling the change in the industry over time: over time, the internal and external environment that each industry faces experience further change. This is likely to cause endogenic differences in the industry at the time series level, and these differences are likely to cause the problem of endogeneity at the corporate governance level. Therefore, while controlling industry and year, this paper further controls the intersection term between industry and year, hoping to reduce the influence.

Instrumental Variable Test: to effectively control the problem of endogeneity, the instrumental variable method was used. Studies have shown that rainfall was negatively correlated with regional pollution levels [69,70] and thus negatively correlated with environmental regulation levels. The rainfall of a region was an exogenous natural phenomenon, which will not have a substantial impact on the green innovation of enterprises. Therefore, we believe that the annual mean precipitation of the enterprise’s location was a more reasonable instrumental variable.

#### 3.3.2. Robustness Test Method

To enhance the robustness of empirical research, we used 3 methods to test the robustness: lag the environmental regulation (ERS) for 1 period, replace the measurement method of green innovation, and replace the measurement method of environmental regulation. The robustness test, the selection of control variables, and the standard error were consistent with the main regression.

Lag environmental regulatory strength (ERS) by 1 period: considering the time lag of macro policies, the variable ERS was put into Equation (1) after lagging for 1 period.

Change the measurement method of green innovation: the number of green innovation applications was used to measure the green innovation of enterprises.

Change the measurement method of environmental regulation: the environmental regulation intensity of different regions was measured by the Urban Pollution Source Regulatory Information Disclosure Index (PIPT), published by the China Institute of Public and Environmental Affairs. The PIPT index mainly measured the strength and quality of the disclosure of pollutant emission information in the region. The higher the environmental quality of the city, the higher the score and the more stringent the environmental regulation policies.

## 4. Empirical Results

### 4.1. Descriptive Statistics

Table 4 presents descriptive results for the major variables. As can be seen from the table, the minimum value of green innovation was 0.693147, and the maximum value was 6.72383, indicating that the level of green innovation of each enterprise was very different. Similarly, the average score for environmental regulation was 13.88765, and the standard deviation was 13.17308, indicating that different enterprises exhibited great differences in terms of environmental regulation.

### 4.2. Baseline Regression Results: Environmental Regulation, and Enterprise Green Innovation

Table 5 presents the results of a baseline regression. Because the selection of control variables itself may affect the significance of empirical results, the size and symbol of the coefficient was kept the same [17,71]. To demonstrate the rigor and robustness of the empirical results of econometrics, we report the gradual addition of control variables. Column (1) gives the regression result of binary regression without any control variables. In columns (2) to (4), three groups of control variables were added; individual enterprise level, infrastructure level, and urban basic characteristics. Column (5) further controls year and industry to obtain the final regression results. The coefficients of the core explanatory variable of all regression results were significantly positive. To some extent, the selection of control variables will not interfere with the empirical results of this paper. The results of the baseline regression show that environmental regulation promotes green innovation, confirming H2.

### 4.3. Endogeneity Test

Panel A in Table 6 reports the regression results after controlling the industry changes over time. From the regression results, we can see that the empirical results were still valid after controlling the industry changes over time. Panel B of Table 6 reports the two-stage regression results of instrumental variables. Column (2) shows that there was a significant negative correlation between the regional average annual rainfall and environmental regulation, which was consistent with the theoretical expectations. The value of the F statistic in the first stage was 34.41, which was much higher than the reference critical value of 8.96 [72]. The possibility of the instrumental variable being weak was not high, and the selection of the instrumental variable was more reasonable. Column (3) shows that the original empirical conclusion was still valid after controlling the endogenous problem.

### 4.4. Robustness Test

Table 7 reports the results of the robustness test. Column (1) is the regression result of one period lag of environmental regulation (ERs), column (2) is the regression result of changing the measurement method of green innovation, and column (3) is the regression result of changing the measurement method of environmental regulation. From the robustness test results, we can see that the empirical results of this paper were robust.

## 5. Further Research: Moderating Effect of City Leader’s Promotion Pressure

Under China’s current governance system, local leaders have greater freedom of speech. The promotion incentive of local leaders will influence their governing choices. Will the promotion incentive or pressure influence their business decisions? Here we discuss the moderating effect of urban leaders’ promotion pressure on environmental regulation and corporate green innovation.

Considering the endogeneity problems that may be caused by the regulation effect, the IV-2SLS method with interaction terms [17] was used in this paper to control the problems of endogeneity. The selection of instrumental variables for environmental regulation was consistent with the above, and the annual average precipitation at the location of the enterprise was adopted.

As for the instrumental variable of official promotion pressure, the tenure of the previous mayor was selected as the instrumental variable in this paper. The duration of the previous mayor’s time in office will have an impact on the current local leaders. The shorter the promotion time of the former mayor, the greater the pressure on his successor, while the promotion pressure of the former mayor will not have a significant impact on the green innovation of the enterprise.

We used the relevant variables in Equation (2) for the regression. Panel A in Table 8 reports the results without controlling any control variables, while Panel B reports the results with further control variables. All the above results indicate that the promotion pressure of city leaders has a further positive moderating effect on the direct positive correlation between environmental regulation and green innovation of enterprises. Thus, our conclusion is that hypothesis H2 is correct.

Panel C in Table 8 reports the regression results of IV-2SLS. Columns (3) and (4) present the regression results of the first stage. As can be seen from the regression results, the tenure of the previous mayor was positively correlated with the promotion pressure of officials, and the regional average annual rainfall was significantly negatively correlated with environmental regulation, which was consistent with the theoretical expectations. The regression results of the instrumental variable method were consistent with the other regression results. Therefore, it can be concluded that the promotion pressure of city leaders has a further positive moderating effect on the direct positive correlation between environmental regulation and green innovation of enterprises, and this conclusion is robust.

## 6. Conclusions

Taking mainland China as the sample, this paper investigated the relationship between the strength of environmental regulation policies, the promotion pressure of urban leaders, and the green innovation of local firms. The basic conclusions of this study are as follows:

(1) Direct policy regulation in the region stimulates the green innovation behavior of enterprises. That is, the strength of environmental regulation is positively correlated with enterprises’ green innovation.
(2) In this paper, three methods were adopted to conduct robustness tests: delaying environmental regulation (ERS) by one period, changing the measurement method of green innovation, and changing the measurement method of environmental regulation. The conclusion of the robustness test was consistent with the basic conclusion, which again verifies that the intensity of environmental regulation was positively correlated with the green innovation of enterprises.
(3) We also addressed the problem of causality identification. To control the potential problem of endogeneity, we further controlled the interaction term between the control industry and the year, which weakened the problem of endogeneity caused by the time series level. Then, the problem of endogeneity was controlled by the tool variable method, which takes the annual mean precipitation of the city as the tool variable. After resolving the problems of endogeneity with these methods, the empirical conclusions remained robust.
(4) We further discussed the moderating effect of urban leaders’ promotion pressure on environmental regulation and corporate green innovation. The results of the empirical study showed that the promotion pressure of city leaders has a further positive moderating effect on the positive correlation between environmental regulation and the green innovation of enterprises. To control the endogeneity of interaction items, an improved 2SLS method was used to test endogeneity. IV-2SLS regression was conducted using the average annual precipitation of the city and the tenure of the previous mayor as instrumental variables of environmental regulation and promotion pressure of local leaders. The regression results support the original empirical conclusion.

To a certain extent, the conclusions of this paper show that the government’s implementation of direct policy regulation can help enterprises complete transformation and upgrading and promotes the sustainable development of enterprises. Moreover, the promotion incentives of local officials also acted as a catalyst. However, based on the existing research conditions, there are still some directions that can be further improved in this paper. For example, there are still some disputes in the academic community about the measurement method of official promotion pressure [29,60]. Based on the availability of data, the research cycle of this paper may not be long enough. The problem of endogeneity control is ongoing. The authors will continue to pay attention to these problems and make improvements in follow-up research.

## 7. Discussion

### 7.1. Implications for Theory and Practice

Enterprise innovation is a multistage process [73] influenced by the internal governance conditions of the enterprise and the external economic and political environment [57]. Under the premise that modern corporate governance and government governance pay more attention to sustainability, it is more meaningful to discuss the problem of enterprise innovation under the framework of the environment–society–governance (ESG). Green innovation of enterprises is the core of sustainable innovation of [10], and environmental governance of the government is the key to sustainable development. It is meaningful to discuss the causal relationships and action paths between them. Emerging market countries, represented by China, are in a period of economic transition, facing the dual pressure of economic and environmental assessment [3]. On the one hand, the government needs to improve the economic performance of enterprises and develop the economy. On the other hand, with the improvement of the economic level, the public’s requirements for governance on the environment also increase. Under the guiding ideology of “federalism with Chinese characteristics”, the dual pressure brought about by local environmental governance and economic development is bound to fall on city leaders [69]. It is necessary to discuss the role of promotion pressure of city leaders in the process of environmental regulation in encouraging the green innovation of enterprises. The vast majority of enterprises’ (green) innovations are based on the microscopic perspective of enterprises [18,19,20,21,22]. In this paper, the impact of the external environment on enterprise innovation is considered at the level of local officials’ demands for political performance for a more in-depth investigation. It is helpful to understand the key to the transformation and optimization of economic growth.

There are a lot of studies on environmental regulations and enterprises’ innovation decision-making. In the early stage, the correlation between them was discussed [34,37], but the causality and mechanism of action were seldom analyzed. With the continuous development of econometrics theory, scholars have gradually begun to discuss the problem of causal identification between the two [22,74], which also provides a good research paradigm for this paper. However, the downside of these studies is that they are more or less subject to econometric controversy. For example, selecting the ventilation coefficient as the instrumental variable [56] without considering the spatial diffusivity, or using causal identification tools such as difference-in-difference to discuss endogeneity cannot fully meet the requirements of this method [18]. In addition, the potential endogeneity problem is often neglected when discussing the moderating effect. Such empirical defects reduce the credibility of the research conclusions. After referring to previous research results, this paper attempts to find more reasonable instrumental variables to achieve a cleaner process of causal identification. The effectiveness and rationality of environmental performance assessment in the process of environmental governance have been debated. Some studies believe that environmental governance brings “compliance costs” to enterprises, increases the operating burden of enterprises, and restrains their innovation activities [67]. Some studies also believe that the pressure of environmental performance appraisal will lead to irrational behavior on the part of city leaders, which will have negative consequences [30]. The conclusions show that the government’s implementation of direct policy regulation can help enterprises complete transformation and upgrading and promotes the sustainable development of enterprises. Moreover, the promotion incentives of local officials also acted as a catalyst. This paper proves the effectiveness of environmental performance appraisal systems in environmental governance from the micro point of view. The microsustainable governance of enterprises and regional sustainable governance are mutually dependent, and they can avoid conflicts and develop together. Governments in economic transition should continue to adhere to the concepts of green development and insist that both economic and environmental issues be tackled.

### 7.2. Limitations and Future Research

This paper also has some shortcomings, which can be addressed by improvements and expansion in the future.

First, there are still some disputes in the academic community about the measurement method of official promotion pressure [29,75]. In addition to the method adopted in this paper, other scholars have considered whether the official is in their second term [43], the age of the official [43,76], and whether there is promotion between the mayor and the municipal party secretary during the term of office [77]. We believe that the above method is flawed. As for whether officials are in the second term, if it is believed that the promotion pressure of officials in the second term is greater than in the first term, it should be assumed that all officials have the opportunity to be promoted to departments with real power, rather than non-organs with real power such as the CPPCC. However, many officials, due to their age, personal background, or the will of their families, no longer have the possibility of being promoted to a department with real power after being promoted to a city leader. Instead, they just want to complete their assessment and retire quietly. On the other hand, more than half of the officials changed their posts directly during or after their first term [29,69]. Therefore, this measure is not sound. A similar problem exists in the measurement of whether the mayor and the municipal party secretary have been promoted during their term of office. The age of officials may be a better indicator. The central idea of this measure is that younger officials are more politically motivated and have a stronger desire for promotion. However, as the Organization Department of the CPC Central Committee and other relevant functional departments put forward the relevant guiding ideology of “promoting cadres with younger ages and higher education”, under the same conditions, younger officials face less promotion pressure. Therefore, the rationality of this measure is also reduced. The above measurement methods are all based on the conditions of officials themselves. However, in the promotion assessment of city leaders by the superior organizational departments, the critical thing is the governance performance. Therefore, we believe that it is reasonable to measure the promotion pressure of local officials based on the governance performance of city leaders. The root cause of the controversy over the reasonableness of the pressure to promote officials is the availability of data. For various economic, political, and other reasons, a lot of data on China’s political relevance are not available. In future research, we plan on using the relevant ideas of machine learning for reference and using cutting-edge economic research methods such as text analysis and public opinion analysis to conduct a more reasonable measurement of the core indicator of official promotion pressure.

Secondly, we have been trying to find a reasonable causality identification method. We tried to use difference-in-difference but were consistently unable to find policy shocks that met the strict exogenous assumption. We also tried the synthetic control method, but the selection of samples in the TREAT and CONTROL groups also failed to meet the relevant assumptions. Thankfully, we found that the instrumental variables we were looking for were reasonable and efficient. In future research, we will closely follow the development of econometrics to solve this problem better.

## Figures and Tables

**Table 1:** Variable definitions.

Variable Symbol	Variable Name	Unit	Variable Definition
green_in	Green innovation	Piece	The natural log of the number of green patents obtained by the firm in that year + 1
ers	Environmental regulation	/	(1) Calculate the environmental pollution emission strength of the i city: $E_{l,\mathrm{it}}=\frac{e_{l,\mathrm{it}}}{Y_{\mathrm{it}}}$ where $e_{l,\mathrm{it}}$ represents the total emissions of the first pollutant in the t period of i city; $Y_{\mathrm{it}}$ Represents the actual gross industrial output value of i city in the t period; $E_{l,it}$ represents the emission strength of the first pollutant in period t of i city. (2) Calculate the environmental pollution emissions strength: ${\hat{E}}_{l,\mathrm{it}}=\sum_{j=1}=\frac{e_{l,\mathrm{it}}}{Y_{\mathrm{it}}}$ (3) Divide one by the other to obtain the environmental pollution emissions’ rela-tive strength: ${\mathrm{ER}}_{l,\mathrm{it}}=\frac{E_{l,\mathrm{it}}}{{\hat{E}}_{l.\mathrm{tt}}}$. (4) Calculate the comprehensive index of environmental regulation of local gov-ernment: ${\mathrm{ER}}_{\mathrm{it}}=1/3\times \left({\mathrm{ER}}_{1,\mathrm{it}}+{\mathrm{ER}}_{2,\mathrm{it}}+{\mathrm{ER}}_{3,\mathrm{tt}}\right)$. (5) Considering the intuitiveness of the sign of regression coefficient, this paper takes the inverse of the index to represent the strength of environmental regulation: $\mathrm{ERS}=1/{\mathrm{ER}}_{\mathrm{it}}$ [3,47].
off_pre	Pressure of promotion	/	(1) 301 prefecture-level cities or regions were divided into 3 levels: 276 ordinary cities (the third level), 19 subprovincial cities and quasi-first-tier cities (the second level), and 6 municipalities and first-tier cities (the first level). For the specific city divisions, see Table 2. (2) Calculate the real GDP growth rate, average wage growth rate, and fiscal surplus of each prefecture-level city. Finally, for ordinary cities, the above three indicators are compared with the weighted average of cities in the province where they are located, which is calculated with real GDP as the weight. For sub-provincial cities, it is compared with the weighted average of all 31 subprovincial cities on GDP growth rate and fiscal surplus. If the weighted average is greater than the current year, the value is 1; otherwise, it is 0. If the unemployment rate is less than that of the current year, it is 1; otherwise, it is 0. (3) The scores were added up to obtain the promotion competitiveness of local officials’ economic growth performance (off_c), the value of which ranged from 0 to 3. The higher the value of the index, the higher the economic performance of local officials, the higher the probability of promotion, and the stronger the promotion competitiveness, the lower the promotion pressure of officials. (4) In order to reflect the promotion pressure of local leaders more intuitively, we added 1 to the value and took the inverse. Namely: off_pre = 1/(1 + off_c).

**Table 2:** City Grade Division.

Grade	Cities
First-grade cities	Beijing, Shanghai, Guangzhou, Shenzhen, Tianjin, and Chongqing (6 in total)
Second-grade cities	Wuhan, Harbin, Shenyang, Chengdu, Nanjing, Xi’an, Changchun, Jinan, Hangzhou, Dalian, Qingdao, Xiamen, Ningbo, Zhengzhou, Changsha, Suzhou, Dongguan, Hefei, and Foshan (19 in total)
Third-grade cities	The others (276 in total)

Note: According to the administrative hierarchy in China and the classification of the economic hierarchy of cities in the Wind database, the 301 cities in this study were divided into three levels. Among them, there were six first-grade cities, including four municipalities directly governed by the central government and first-tier cities (Shenzhen and Guangzhou). There were 19 s-grade cities, including subprovincial cities (except Shenzhen and Guangzhou) and new first-tier cities. There were 276 third-grade cities, which account for the rest.

**Table 3:** Variable definitions.

Variable Symbol	Variable Name	Unit	Variable Definition
establish	Time of establishment of the enterprise	Year	Current time year minus year of establishment
share	Management shareholding ratio	%	Sum of the number of shares held by the executive team, divided by all shares
top	The shareholding ratio of the top shareholder	%	The shareholding ratio of the largest shareholder in that year
drcnum	Management size	People	Total number of directors for the year
lnassets	Total assets	Million yuan	The natural log of the number of total assets
roa	Return on assets	%	Net profit divided by total assets
lev	Debt to asset ratio	%	Total liabilities divided by total assets
lnschool	School number	Place	The natural log of the number of schools
lnmobile	Number of mobile phones	Ten thousand households	The natural logarithm of the number of mobile phone subscribers per 10,000 people at the end of the year
lnhospital	Amount of III-A general hospitals	Place	The natural log of the number of III-A general hospitals
lnpgdp	Real GDP per capita	Yuan	The natural log of real GDP per capita
lnindout	Degree of industrialization	Ten thousand yuan	The natural logarithm of the total industrial output value above the designated level
thrind	Proportion of tertiary industry	%	Output value of tertiary industry divided by GDP
lninvest	Fixed asset investment	Ten thousand yuan	The natural log of the number of fixed asset investment

**Table 4:** Descriptive statistics.

Variable	Obs	Mean	Std. Dev.	Min	Max
green_in	6813	1.440076	0.784879	0.693147	6.72383
ers	6813	13.88765	13.17308	0.0951866	71.25961
establish	6813	18.36711	5.150727	7	34
share	6813	6.22217	12.37154	0	61.1109
top	6813	34.81655	14.5737	8.48	74.96
drcnum	6813	9.434857	2.564274	4	18
lnassets	6813	9.735189	0.5669104	8.46253	11.704
roa	6813	0.0402027	0.0489199	−0.257159	0.206069
lev	6813	0.4455504	0.1963248	0.047948	0.828076
lnmobile	6813	6.770936	0.8387994	3.178054	8.296047
lnschool	6813	5.51414	0.5371717	2.302585	6.411819
lnhospital	6813	2.373694	5.371458	0	58
lnpgdp	6813	11.4913	0.4754829	9.26568	15.67181
lnindout	6813	9.194338	1.03648	2.031117	10.45857
thrind	6813	54.22762	14.14914	14.36	81
lninvest	6813	8.120313	0.7962099	3.583897	9.766554
lngreen	6813	1.52585	0.8615622	0.693147	6.07764

**Table 5:** Baseline results: environmental regulation and corporate green innovation.

Variables	(1)	(2)	(3)	(4)	(5)
ers	0.0028244 ***	0.0030615 ***	0.0053525 ***	0.0035263 **	0.0057763 ***
	[3.31]	[3.68]	[3.93]	[2.42]	[3.82]
establish		−0.0056531 **	−0.0108075 ***	−0.010178 ***	−0.0108998 ***
		[−2.58]	[−3.73]	[−3.51]	[−3.52]
share		0.0007638	−0.0000476	0.001103	0.0005564
		[0.93]	[−0.05]	[1.03]	[0.51]
top		0.0010838	−0.0005307	−0.0006491	−0.0007992
		[1.38]	[−0.57]	[−0.67]	[−0.79]
drcum		0.0059508	0.0037781	−0.0003318	0.0017207
		[1.35]	[0.73]	[−0.06]	[0.31]
lnassets		0.3344237 ***	0.3527234 ***	0.2901184 ***	0.329044 ***
		[12.01]	[9.66]	[7.64]	[8.14]
roa		0.3810581 *	0.6145153 **	0.7102136 **	0.7457738 **
		[1.67]	[2.25]	[2.27]	[2.28]
lev		0.1147679	0.132005	0.1351266	0.1603578 *
		[1.64]	[1.57]	[1.50]	[1.76]
lnmobile			−0.078403 **	−0.1251364 **	−0.135748 **
			[−2.13]	[−2.29]	[−2.71]
lnschool			0.0698884	0.112339 *	0.0875084
			[1.49]	[1.90]	[1.43]
lnhospital			0.0570487	0.0094339	0.0469903
			[1.21]	[0.16]	[0.75]
lnpgdp				0.035135	−0.0283285
				[0.69]	[−0.5]
lnindout				0.0372308	0.0180994
				[1.08]	[0.48]
thrind				0.0051302 **	0.0041532 *
				[2.38]	[1.71]
lninvest				0.0127518	0.0043786
				[0.29]	[0.1]
*R* ^2^	0.0022	0.0764	0.1369	0.0537	0.1390
Year	No	No	No	No	Yes
Industry	No	No	No	No	Yes
*N*	6813	6813	6813	6813	6813

Note: T statistics in brackets. *** *p* < 0.01, ** *p* < 0.05, * *p* < 0.1.

**Table 6:** Endogeneity test results.

Variables	Panel A	Panel B	
	(1)	(2)	(3)
rain		−0.0035171 ***	
		[−8.91]	
ers	0.0053221 ***		0.0304982 **
	[3.33]		[2.17]
Constant	−2.741581 ***	−88.83805 ***	−5.237885 ***
	[−3.19]	[−8.55]	[−2.62]
Controls	Yes	Yes	Yes
Industry	Yes	Yes	Yes
Year	Yes	Yes	Yes
Industry × Year	Yes	No	No
C-D Wald F stat		34.41 ***	
*R* ^2^	0.2116	0.3136	
*N*	6813	6813	6813

Note: T statistics in brackets. Due to space limitations, the details of control variables are not listed. *** *p* < 0.01, ** *p* < 0.05.

**Table 7:** Robustness test results.

Variables	(1)	(2)	(3)
ers	0.0043364 ***	0.0038179 ***	
	[2.68]	[2.88]	
pipt			0.0053887 **
			[2.29]
Controls	Yes	Yes	Yes
*R* ^2^	0.1394	0.1734	0.1315
Year	Yes	Yes	Yes
Industry	Yes	Yes	Yes
*N*	4132	6813	6813

Note: T statistics in brackets. Due to space limitations, the details of control variables are not listed. *** *p* < 0.01, ** *p* < 0.05.

**Table 8:** Results of moderating effect of city leader’s promotion pressure.

Variables	Panel A	Panel B	Panel C (IV-2SLS)		
	(1)	(2)	(3)	(4)	(5)
ers	0.0031665 **	0.0054071 ***			0.0312304 ***
	[2.28]	[3.74]			[4.23]
off_pre	0.1360055	0.0445764			0.0318533
	[0.94]	[0.71]			[0.32]
ers × off_pre	0.0102087 ***	0.0108314 ***			0.042215 **
	[1.51]	[3.50]			[3.06]
rain				−0.02356 ***	
				[−6.21]	
tenure			0.00637 ***		
			[5.13]		
Controls	No	Yes	Yes	Yes	Yes
Year	No	Yes	Yes	Yes	Yes
Industry	No	Yes	Yes	Yes	Yes
F statistics			21.32 ***	33.34 ***	
*R* ^2^	0.0535	0.1389	0.0721	0.2971	
*N*	6813	6813	6813	6813	6813

Note: T statistics in brackets. Due to space limitations, the details of control variables are not listed. *** *p* < 0.01, ** *p* < 0.05.

## Data Availability

All data can be obtained by email from the corresponding author.
